# Reducing state anxiety with alpha-frequency transcranial alternating current stimulation

**DOI:** 10.21203/rs.3.rs-8756192/v1

**Published:** 2026-02-03

**Authors:** Rubén Romero-Marín, Simon Fankhauser, Javier Solana-Sánchez, Ruben Perellón-Alfonso, Josep Maria Tormos-Muñoz, Luiz Pessoa, David Bartrés-Faz, Davide Cappon, Álvaro Pascual-Leone, Gabriele Cattaneo

**Affiliations:** Institut Guttmann Institut Universitari de Neurorehabilitació adscrit a la UAB, Badalona, Spain; Institut Guttmann Institut Universitari de Neurorehabilitació adscrit a la UAB, Badalona, Spain; Institut Guttmann Institut Universitari de Neurorehabilitació adscrit a la UAB, Badalona, Spain; Institut Guttmann Institut Universitari de Neurorehabilitació adscrit a la UAB, Badalona, Spain; Centro de Investigación Traslacional San Alberto Magno, Universidad Católica de Valencia San Vicente Mártir, Valencia, Spain; Department of Psychology, University of Maryland, College Park, MD, USA; Institut d’Investigacions Biomèdiques August Pi I Sunyer (IDIBAPS), Barcelona, Spain; Department of Neurology, Harvard Medical School, Boston, MA, USA; Hinda and Arthur Marcus Institute for Aging Research and Deanna and Sidney Wolk Center for Memory Health, Hebrew SeniorLife, Boston, MA, USA; Institut Guttmann Institut Universitari de Neurorehabilitació adscrit a la UAB, Badalona, Spain

**Keywords:** tACS, resilience, anxiety, stress

## Abstract

**Background:**

Anxiety reactivity to acute stress is a transdiagnostic vulnerability factor. We tested whether a single session of alpha-frequency transcranial alternating current stimulation (tACS) targeting the frontoparietal control network reduces stress-evoked state anxiety in healthy adults.

**Methods:**

In a randomized, blinded, sham-controlled study, 42 participants (mean age 58.9 years) completed an acute stress task before and after stimulation. The task was an adapted moving-circles paradigm in which circle collisions triggered a brief aversive event (mild electric shock plus unpleasant noise and a white flash). Active stimulation consisted of 20 min of 10-Hz tACS (2.0 mA/channel; 30-s ramp up/down) delivered via electrodes at F3, P3, Cz, and T7 (0° phase at F3/P3; 180° at Cz/T7). Sham stimulation used the same montage and ramp periods but no sustained current.

**Results:**

State anxiety showed a significant Time × Protocol interaction (F(1,35)=4.22, p=0.047): STAI-S decreased after active tACS (Δ=−3.16) but increased slightly after sham (Δ=+1.17). Perceived stress appraisal (SAAS) did not change. Resting-state alpha power at F3/P3 showed no reliable pre–post effects. During the task, left-frontal relative alpha differed by protocol and showed a trend toward larger increases following active tACS. Electrodermal and pupil indices changed across sessions in both groups, with no differential stimulation effects.

**Conclusions:**

A single alpha-tACS session produced a modest, selective reduction in stress-evoked state anxiety, supporting oscillatory neuromodulation as a scalable approach to dampen anxiety reactivity.

## Introduction

Mental health disorders, particularly anxiety and depressive disorders, affect a substantial proportion of the global population and represent a major public health challenge (Cheng et al., 2024; J. Liu et al., 2024; Santomauro et al., 2021). Chronic stress has been consistently identified as one of the most relevant environmental risk factors contributing to the onset, maintenance, and severity of these conditions. Exposure to sustained psychological, social, or physical stressors leads to persistent activation of the hypothalamic–pituitary–adrenal (HPA) axis, resulting in prolonged cortisol secretion and widespread neurobiological consequences (Chrousos, 2009; Herbert, 2013; Pariante & Lightman, 2008). Although acute stress responses are adaptive, chronic dysregulation of the stress system is associated with structural and functional alterations in brain regions critical for emotion regulation, including the prefrontal cortex, hippocampus, and amygdala (McEwen, 2004; Sapolsky, 2000).

Beyond neuroendocrine alterations, chronic stress exerts profound effects on immune function, promoting a state of low-grade systemic inflammation characterized by increased levels of proinflammatory cytokines such as interleukin-6, tumor necrosis factor-α, and C-reactive protein (Dantzer et al., 2008; Miller & Raison, 2016; Rohleder, 2014). These inflammatory mediators can access the brain through multiple pathways, activate microglia, and disrupt synaptic plasticity, neurotransmission, and neurogenesis, thereby contributing to affective and cognitive symptoms (Calcia et al., 2016; Capuron & Miller, 2011; A. Cattaneo et al., 2015). Dysregulation of stress–immune interactions has been strongly implicated in the pathophysiology of depression and anxiety and is associated with poorer clinical outcomes and increased vulnerability to relapse (Malhi & Mann, 2018; Slavich & Irwin, 2014).

Beyond their role as symptoms, heightened anxiety and maladaptive stress responses constitute core vulnerability factors that increase risk for the onset and persistence of common mental disorders. Elevated anxiety—particularly when expressed as exaggerated reactivity to threat and impaired recovery—has been linked to broader internalizing psychopathology and poorer functional outcomes. Likewise, chronic stress exposure and sustained stress-system activation can amplify negative affect, bias threat appraisal, and interfere with cognitive control, thereby contributing to the development and maintenance of anxiety and depressive disorders. Together, anxiety and stress-related dysregulation form a transdiagnostic pathway through which vulnerability is expressed and reinforced over time.

Importantly, not all individuals exposed to comparable stressors develop psychopathology. This observation has led to increasing interest in the concept of stress resilience, broadly defined as the capacity to maintain or rapidly regain psychological stability under adversity, in part by dampening excessive anxiety responses and supporting efficient stress recovery (Faye et al., 2018). From a neurobiological perspective, resilience reflects the efficient regulation of stress responses, including faster recovery of cortisol levels, enhanced parasympathetic activity, and effective top-down control exerted by prefrontal regions over limbic circuits (Madison, 2021; Notebaert et al., 2024). Higher resilience has been associated with reduced amygdala reactivity, greater prefrontal engagement, and stronger fronto-limbic functional connectivity, supporting adaptive emotional regulation and cognitive flexibility under stress (Castelo-Branco & Fregni, 2020; Faye et al., 2018).

Given the central role of prefrontal–limbic networks in stress regulation, non-invasive brain stimulation (NIBS) techniques have emerged as promising tools to modulate neural circuits implicated in resilience. While much of the existing literature has focused on transcranial direct current stimulation, converging evidence indicates that NIBS can influence autonomic markers of stress, HPA-axis activity, and emotional regulation, suggesting potential utility not only for symptom reduction but also for preventive and resilience-enhancing interventions (Guo et al., 2023; Ren et al., 2025). Consistent with this view, our earlier findings suggest that prefrontal neuromodulation may attenuate stress appraisal (Romero-Marín et al., 2026). Among these techniques, transcranial alternating current stimulation (tACS) offers a mechanistically distinct approach by targeting neural oscillations rather than inducing tonic changes in cortical excitability. tACS delivers sinusoidal alternating currents that interact dynamically with endogenous brain rhythms, enabling frequency- and phase-specific modulation of neural activity (Helfrich et al., 2014; Polania et al., 2021). By entraining oscillatory activity within targeted networks, tACS can influence functional connectivity and information flow across distributed circuits involved in cognitive control, emotional regulation, and stress processing (Reinhart & Nguyen, 2019; Saturnino et al., 2017).

Neural oscillations play a fundamental role in coordinating prefrontal–limbic interactions, which are critical for adaptive stress responses. Modulation of oscillatory synchrony through tACS has been shown to induce sustained changes in cortical dynamics, likely mediated by neuroplastic mechanisms involving NMDA receptor activity (Wischnewski et al., 2019). Furthermore, recent evidence suggests that tACS may influence neuroinflammatory processes and microglial activation, particularly when applied at gamma frequencies, highlighting a potential pathway through which oscillatory neuromodulation could impact stress-related neurobiological mechanisms (Guo et al., 2023).

Despite these promising findings, evidence remains limited regarding whether brief, scalable tACS interventions can modulate anxiety responses elicited by acute stress in healthy adults—a mechanistic target relevant to transdiagnostic vulnerability. This question is particularly important for preventive approaches aimed at reducing vulnerability by dampening stress-related anxiety reactivity and supporting adaptive regulation. Here, we tested whether a single session of alpha-frequency tACS targeting nodes of the frontoparietal control network (FPCN) modulates state anxiety and perceived stress appraisal following an acute laboratory stressor, compared with sham stimulation. As secondary outcomes, we examined exploratory neurophysiological and autonomic indices acquired at rest and during task exposure.

## Materials and methods

### Participants

Participants were healthy adults recruited from the Barcelona Brain Health Initiative (BBHI) cohort, a longitudinal study focused on factors related to brain health and resilience across adulthood (G. Cattaneo et al., 2020). Eligibility criteria were defined to ensure participant safety and to minimize potential confounding factors affecting neurophysiological responses to stress and neuromodulation.

Exclusion criteria included a history of neurological, psychiatric, or cardiovascular disorders, as well as any contraindication to non-invasive brain stimulation (NIBS). Specifically, individuals were excluded if they reported intracranial metallic implants, implanted electronic devices (e.g., pacemakers, vagus nerve stimulators, cochlear implants), a history of seizures or epilepsy, structural brain lesions, brain tumors, or any other medical condition associated with a reduced seizure threshold. Additional exclusion criteria comprised recent use of psychoactive substances, sleep deprivation, excessive consumption of alcohol or caffeine, and the use of medications with known proconvulsant effects. In accordance with international safety recommendations for NIBS, pregnant participants were also excluded (Rossi et al., 2021).

All participants provided written informed consent after receiving a detailed explanation of the study procedures. The study protocol was approved by the Clinical Research Ethics Committee with Medicines of the Fundació Unió (CEIm; approval code CEI 23/35) and was registered at ClinicalTrials.gov (NCT06051071).

The final sample for the tACS intervention (see Table 1) consisted of 42 participants (16 women), with a mean age of 58.92 years (SD = 7.49). Participants were randomly assigned to one of two experimental conditions: active tACS (n = 22) or sham tACS (n = 20). All participants completed both the pre- and post-stimulation assessments, and no participant was excluded after randomization.

### Procedures

**Figure 1:**
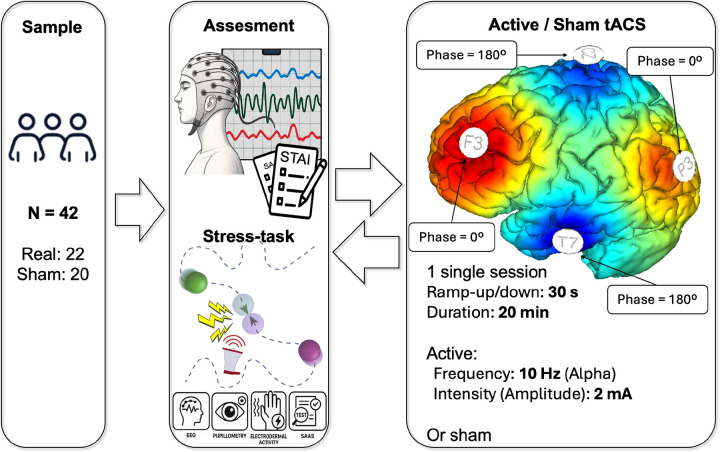
Study design. Participants completed a baseline assessment followed by the acute stress task, and then received a tACS session (active or sham). The figure also depicts a simulation of the electric field distribution during active tACS using the specified stimulation parameters. After stimulation, participants repeated the same assessment and stress task.

Our primary aim was to assess behavioral changes in state anxiety and stress response before and after stimulation. Participants completed a baseline assessment followed by an acute stress task, received the tACS intervention, and subsequently underwent a post-stimulation assessment and a second exposure to the same stress task (see Fig. 1).

### Primary outcomes: Behavioral changes in perceived stress and anxiety levels induced by acute stressor paradigm

Pre and post assessments were conducted to assess the impact of the tACS stimulation protocol on anxiety and perceived stress. Participants were evaluated on an adapted version of the moving-circles paradigm (Limbachia et al., 2021; Meyer et al., 2019), where two circles move around the screen, sometimes moving closer and at times moving away from each other. When the circles touch, participants are delivered a mild electric stressor. In our adaptation, participants were seated in a chair and instructed to focus on a black screen displaying the two circles moving across it. When the circles collided, a distressing event was triggered, involving a brief electric discharge to the hand accompanied by an unpleasant noise and a white flash on the screen. The duration of this stressor event ranged from 0.2 to 2 seconds. Electric discharges were administered using a Digitimer Constant Current Stimulator DS7A (Digitimer Ltd) through two electrodes (input and output) placed on the right hand.

Psychophysiological stress responses were assessed during the task using pupillometry and EDA. EDA were recorded using Biopac MP150 hardware (Biopac Systems Inc), with sensors placed on the second and third fingers of the left hand. Pupillometry data were collected using a Tobii Pro Nano Eye Tracker (Tobbi AB). The sampling rate was 1000 Hz for EDA and 60 Hz for pupillometry.

Data preprocessing was performed using the same pipeline and parameters described in detail in our previous study (Romero-Marín et al., 2026).

The entire task lasted for 7 minutes, during which participants were instructed to maintain their gaze on the screen and minimize blinking to facilitate accurate pupillometry measurements.

This task was administered both before the commencement and upon completion of tACS session. Perceived stress and anxiety levels were evaluated immediately after the task using the post task subscale of the Stress Appraisals of Acute Stress (SAAS) scale (Mendes et al., 2007), as well as the state subscale (STAI-S) of the State-Trait Anxiety Inventory (STAI). The SAAS scale has been developed to be used in laboratory stress paradigms and measure demand and resource appraisals. The post task subscale is composed by 10 items and participants responded using a 7-point likert scale ranging from 1 to 7. Higher scores represent higher stress appraisal. The STAI state subscale, designed for clinical use, comprises 20 items. Participants respond using a 4-point Likert scale ranging from 0 to 3. Higher scores represent higher state anxiety.

### tACS Protocol

tACS was delivered using a Starstim tES device (Neuroelectrics^®^, Barcelona, Spain) controlled via the NIC2 software (v1.4.0). Stimulation was administered through four circular sponge electrodes (Sponstim, 8 cm^2^) integrated into the same EEG cap used for signal acquisition. Prior to stimulation onset, electrode–skin contact quality was verified, and stimulation was initiated only when impedance values for all channels were within acceptable limits, as indicated by the NIC2 impedance check.

The stimulation montage was designed to modulate the frontoparietal control network (FPCN), a circuit critically involved in cognitive control and stress regulation (Cole et al., 2014; He et al., 2024; Scult et al., 2019). Electrodes were positioned at F3, P3, Cz, and T7, corresponding to frontal, parietal, central, and temporal nodes of the network (see Fig. 1). In the active tACS condition, a sinusoidal current at 10 Hz (alpha frequency) was applied with an intensity of 2.0 mA per channel. Phase configuration was set to 0° at F3 and P3 (in-phase stimulation) and 180° at Cz and T7 (anti-phase stimulation), resulting in synchronous alpha-frequency stimulation between frontal and parietal sites. Stimulation onset and offset were ramped using 30-second ramp-up and ramp-down periods to minimize transient sensations.

In the sham condition, the same electrode montage and ramp periods were applied, but no sustained current was delivered after the initial ramp-up, ensuring effective blinding of participants to stimulation condition.

Each stimulation session lasted 20 minutes. During stimulation, participants remained seated comfortably and were instructed to maintain visual fixation on a black screen with a central fixation cross, while minimizing head and body movements. No cognitive or behavioral task was performed during stimulation to avoid potential confounding effects and to isolate the neuromodulatory impact of tACS on subsequent stress responses.

To initiate stimulation, the EEG recording device was temporarily disconnected and replaced with the Starstim stimulator while maintaining electrode positioning. After completion of the stimulation session, the Starstim EEG device was disconnected, EEG recording was re-established, and post-stimulation assessments were conducted following the same procedures as the baseline phase.

Throughout the stimulation session, impedance values were continuously monitored via NIC2 to ensure stable current delivery and participant comfort. All stimulation parameters were selected in accordance with established safety guidelines for non-invasive brain stimulation.

### Statistical analysis

Changes in self-reported state anxiety and perceived stress appraisal were examined by comparing pre- and post-intervention scores on the STAI-S and SAAS using the Wilcoxon signed-rank test. All statistical analyses and data visualizations were conducted in JASP (JASP Team, 2019).

To test intervention-related effects on physiological outcomes and model-derived indices, we ran a set of repeated-measures ANOVAs assessing within-subject changes from Pre to Post stimulation across dependent variables derived from pupillometry and electrodermal activity (EDA).

Physiological measures (pupil and EDA amplitudes). Anticipatory autonomic responses were operationalized as pupil amplitude and EDA amplitude, quantified as the area under the curve (AUC) in the seconds immediately preceding each event. These raw amplitude measures were entered into repeated-measures ANOVAs to evaluate time-dependent effects.

Model fit indices (R^2^ and beta estimates). To characterize stimulus–response coupling, linear regression models were fitted between each physiological signal and a continuous stimulus proximity trace. For each participant and condition, model fit was summarized using the coefficient of determination (R^2^), reflecting the proportion of variance in the physiological signal accounted for by the stimulus. Beta estimates (EST) were extracted to index the direction and magnitude of the association. Both R^2^ and EST were analyzed with repeated-measures ANOVAs to assess pre-to-post changes.

Model accuracy (mean squared error). Model accuracy was quantified using the mean squared error (MSE). For EDA-derived MSE values, a log transformation was applied prior to inferential testing to improve normality. Repeated-measures ANOVAs were then used to evaluate changes across time points.

For each ANOVA, we report sums of squares (SS), degrees of freedom (DF), mean squares (MS), F statistics, uncorrected p values (p-unc), and generalized eta squared (η^2^g) as an effect size estimate.

Where applicable, the sphericity correction factor (ε) is also reported.

## Results

### Sample and protocol completion

**Figure 2:**
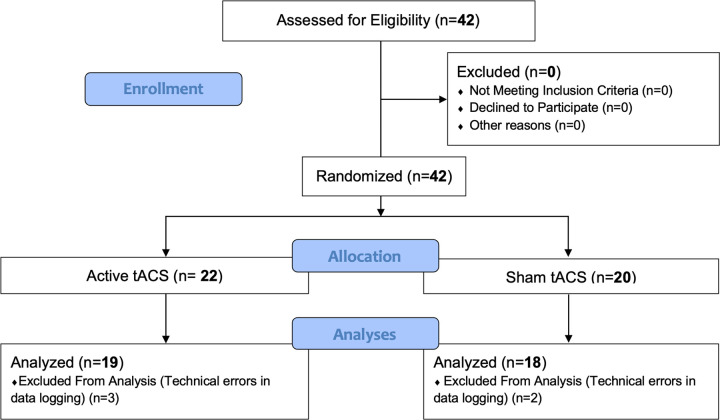
Study flow diagram following CONSORT guidelines (Schulz et al., 2010).

A total of 42 participants completed the tACS study. No participant withdrew from the study and none were discontinued for clinical reasons. However, five participants were excluded from the final analyses due to technical incidents affecting neurophysiological acquisition, including synchronization mismatches between the experimental task and the LSL stream, intermittent incompatibilities with the BIOPAC system, and network interruptions compromising multimodal data integration (see Fig. 2).

### Perceived stress and anxiety

#### State anxiety (STAI-S)

For state anxiety, a repeated-measures ANOVA was conducted with Time (pre vs. post) as the within-subject factor and Protocol (active tACS vs. sham) as the between-subject factor. The main effect of Time was not significant, F(1, 35) = 0.895, p = .351, indicating no overall reduction in state anxiety from pre- to post-intervention when collapsing across groups. In contrast, the Time × Protocol interaction was significant, F(1, 35) = 4.222, p = .047, η^2^p = .108 (MS_error = 20.47), demonstrating that pre–post changes differed between conditions.

**Figure 3:**
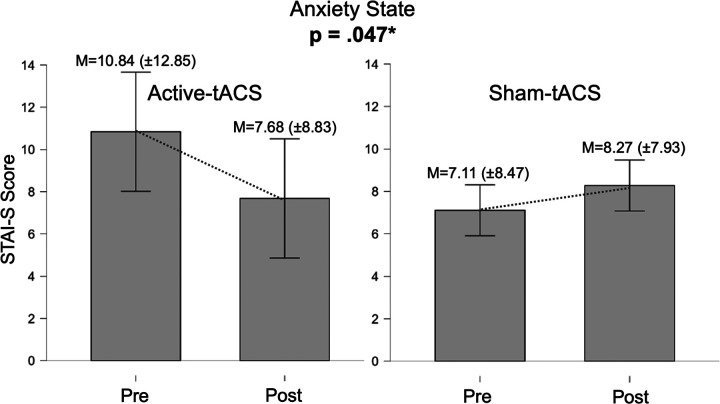
Comparative bar plots for the STAI-S. The figure shows a decrease in STAI-S scores in the active tACS group and a slight increase in the sham group, indicating a significant Time × Protocol interaction.

Descriptive statistics supported this pattern. In the active tACS group (n = 19), STAI-S scores decreased from M = 10.842 (SD = 12.855) to M = 7.684 (SD = 8.838) (Δ = −3.158). In the sham group (n = 18), scores increased slightly from M = 7.111 (SD = 8.478) to M = 8.278 (SD = 7.932) (Δ = +1.167). The resulting difference-in-differences (Δ_active − Δ_sham = − 4.325) was consistent with the significant interaction and favored active tACS in terms of change in state anxiety (see Fig. 3).

#### Perceived stress appraisal (SAAS)

The same repeated-measures ANOVA approach (Time: pre vs. post; Protocol: active vs. sham) was applied to SAAS scores. Neither the main effect of Time nor the Time × Protocol interaction was significant (Time: F(1, 35) = 0.220, p = .642, η^2^p ≈ .006; Time × Protocol: F(1, 35) = 0.128, p = .723, η^2^p ≈ .004; MS_error = 0.015).

Descriptively, SAAS remained essentially unchanged in the active group (n = 19; M = 0.512 (SD = 0.264) pre; M = 0.509 (SD = 0.194) post) and showed a similarly small shift in the sham group (n = 18; M = 0.538 (SD = 0.353) pre; M = 0.514 (SD = 0.350) post). Overall, these results do not provide evidence for pre–post changes in perceived stress appraisal or differential effects between stimulation conditions.

### Resting-state EEG

Pre–post comparisons of resting-state EEG did not show significant changes in alpha power at individual electrodes. Specifically, alpha power at F3 was not significantly different between sessions (t(36) = 0.243, p = .595; Wilcoxon z = 0.596, p = .725), and alpha power at P3 also did not differ significantly (t(36) = − 1.182, p = .123; Wilcoxon z = − 0.249, p = .406).

**Figure 4:**
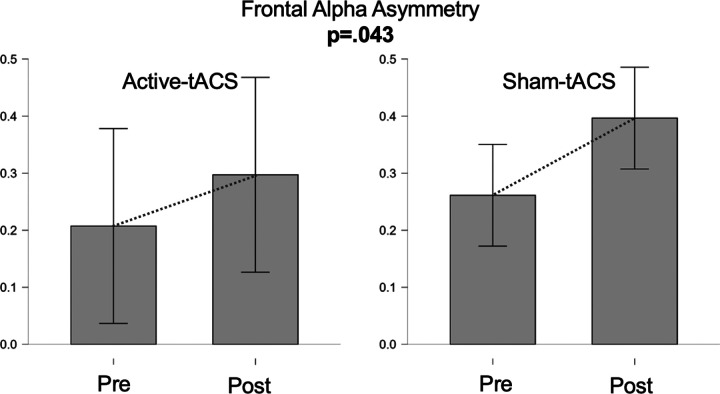
FAA results from Study. Mean FAA scores are compared before and after treatment for both experimental conditions.

In contrast, frontal alpha asymmetry (FAA) increased from pre to post (see Fig. 4). Because the distribution of paired differences showed a deviation from normality (Shapiro–Wilk W = 0.780, p < .001), the non-parametric test was prioritized and indicated a significant increase (Wilcoxon V = 237.000, z = − 1.727, p = .043; alternative: FAA_pre < FAA_post). When FAA was examined using a repeated-measures ANOVA with Time (pre vs. post) and Protocol (active vs. sham), neither the main effect of Time (F(1, 35) = 2.919, p = .096) nor the Time × Protocol interaction (F(1, 35) = 0.120, p = .731) reached statistical significance, suggesting that—under this model—the magnitude of FAA change was not reliably different between protocols.

### Task-based EEG

Task-based EEG analyses focused on relative alpha power in the left frontal region (F3) using a repeated-measures model with a within-subject factor (pre vs. post) and Protocol (active vs. sham) as a between-subject factor. The repeated-measures ANOVA showed a trend toward a within-subject effect that did not reach conventional significance (p = .088) and no significant interaction between the within-subject factor and Protocol (p = .289). In contrast, the between-subjects effect of Protocol was significant (F(1, 32) = 4.510, p = .042), indicating overall group differences in left frontal alpha power during the task.

To further characterize pre–post changes, the change score in relative left frontal alpha power (Δ) was compared between groups. Normality assumptions were met (Shapiro–Wilk W = 0.986, p = .932). An independent-samples t-test (directional hypothesis: active > sham) indicated a trend toward greater increases in the active group (t(33) = 1.386, p = .088, d = 0.469), with mean change M = 2.931 (SD = 4.957, n = 17) for active tACS and M = 0.567 (SD = 5.123, n = 18) for sham. A consistent trend was observed with the Mann–Whitney test (U = 197.0, p = .076). Overall, these findings suggest a tendency toward increased left frontal alpha power during the task following active tACS relative to sham, although group differences in change scores did not reach the conventional p < .05 threshold.

### Electrodermal activity (EDA)

EDA outcomes were analyzed using repeated-measures ANOVAs with Time (pre vs. post) as a within-subject factor and Condition (active vs. sham) as a between-subject factor (see Table 2), focusing on indices derived from the EDA–stimulus model (R^2^, log MSE, and beta estimates).

A significant main effect of Time was found for R^2^ (F = 4.582, p = .041, η^2^g = 0.051), indicating an overall change in model fit from pre to post regardless of stimulation condition. There were no significant effects of Condition (F = 1.021, p = .321) and no Time × Condition interaction (F = 0.010, p = .923).

For log-transformed MSE, a strong main effect of Time was observed (F = 31.514, p < .001, η^2^g = 0.154), consistent with improved model accuracy (lower error) post-intervention across participants. Neither Condition (F = 0.155, p = .697) nor the interaction (F = 0.001, p = .980) was significant. Similarly, beta estimates (EST) showed a significant main effect of Time (F = 13.626, p = .001, η^2^g = 0.102), reflecting a global pre–post change in the stimulus–EDA relationship. No significant effects of Condition (F = 2.123, p = .156) or Time × Condition interaction (F = 2.765, p = .107) were detected. Taken together, EDA results indicate reliable pre–post changes in model-derived parameters with small-to-moderate effect sizes, but these changes were comparable in the active and sham conditions, providing no evidence for a differential tACS effect on EDA indices.

### Pupil diameter

Pupillometry outcomes were analyzed using the same repeated-measures ANOVA structure (Time: pre vs. post; Condition: active vs. sham), focusing on model-derived indices (R^2^, MSE, and beta estimates; see Table 2).

For pupil R^2^, the main effect of Time was significant (F = 4.385, p = .045, η^2^g = 0.032), suggesting a modest overall change in pupil–stimulus model fit from pre to post across conditions. The effect of Condition did not reach significance (F = 2.988, p = .095) and there was no Time × Condition interaction (F = 0.005, p = .942).

For pupil MSE, no significant effects were observed for Time (F = 0.392, p = .536), Condition (F = 0.283, p = .599), or their interaction (F = 0.902, p = .350), indicating stable model error across sessions and groups. For pupil beta estimates, the main effect of Time was significant (F = 5.313, p = .029, η^2^g = 0.035), reflecting a pre–post change in the stimulus–pupil association. However, neither the Condition effect (F = 2.540, p = .122) nor the Time × Condition interaction (F = 0.156, p = .696) was significant. Overall, pupillometry results reveal modest pre–post changes in model fit and slope parameters, but no evidence that active tACS produced differential effects relative to sham.

## Discussion

The present study aimed to examine whether a single session of alpha-frequency tACS applied over prefrontal regions could modulate behavioral, neurophysiological, and autonomic responses to an acute stressor in a healthy adult sample. Specifically, we investigated changes in state anxiety and perceived stress, as well as EEG- and autonomic-based indices of stress reactivity, using a well-established laboratory stress paradigm.

At the behavioral level, the main finding of the tACS study was a significant Time × Protocol interaction for state anxiety, as measured by the STAI-S. Participants receiving active tACS showed a reduction in state anxiety following stimulation, whereas those in the sham condition exhibited a slight increase. Although absolute anxiety levels were low in both groups—consistent with the inclusion of psychologically healthy participants—this pattern suggests a specific acute effect of tACS on emotional reactivity to stress.

This result is in line with previous work showing that prefrontal neuromodulation can attenuate anxiety-related responses, particularly in non-clinical populations where baseline symptom levels are low and effect sizes are therefore expected to be modest (Chen et al., 2024; Clancy et al., 2018). From a mechanistic perspective, alpha-frequency tACS targeting frontoparietal networks may facilitate top–down regulatory control over limbic stress responses, thereby reducing transient anxiety states elicited by acute stress exposure.

In contrast, perceived stress appraisal, assessed with the SAAS, did not show significant pre–post changes nor differential effects between active and sham stimulation. This dissociation between state anxiety and cognitive appraisal of stress has been reported in prior stress research, where affective and autonomic responses may change without corresponding shifts in cognitive evaluations of the stressor (Shiban et al., 2016). Cognitive appraisals are generally considered more stable and may be less sensitive to single-session interventions, particularly in healthy individuals.

Interestingly, this pattern differs from our previous study using repeated home-based tDCS, in which a reduction in perceived stress appraisal (SAAS) was observed following multiple stimulation sessions, although without a sham control group (Romero-Marín et al., 2026). Taken together, these findings suggest a potential dissociation between acute and cumulative effects of neuromodulation: while a single session of tACS may preferentially modulate affective state anxiety, repeated neuromodulation protocols may be required to induce measurable changes in stress-related cognitive appraisals.

Resting-state EEG analyses revealed no significant pre–post changes in local alpha power at frontal (F3) or parietal (P3) electrodes. A modest increase in FAA was observed when collapsing across protocols, but this effect did not survive the factorial repeated-measures ANOVA and therefore cannot be unequivocally attributed to active tACS.

FAA has been proposed as a marker of approach–avoidance motivation and emotional vulnerability (Davidson, 1998), with some studies suggesting that greater left-sided frontal activation may be associated with resilience-related traits. However, recent meta-analyses indicate that FAA effects in depression and stress-related psychopathology are small, heterogeneous, and methodologically sensitive (Kołodziej et al., 2021; Luo et al., 2025; van der Vinne et al., 2017). In this context, the nonspecific FAA increase observed here is more parsimoniously explained by unspecific experimental factors, such as task repetition, habituation, or post-task relaxation, rather than by a direct neuromodulatory effect.

Notably, a similar absence of robust FAA modulation was also observed in our previous HB-tDCS study, suggesting that resting-state alpha asymmetry may be insufficiently sensitive to capture subtle neuromodulatory effects in healthy samples. High intraindividual variability in resting EEG measures further complicates detection of small effects, particularly in moderately sized samples (E. Liu et al., 2024).

In contrast to resting-state measures, task-based EEG analyses revealed a more suggestive pattern. Relative alpha power at the left frontal electrode (F3) showed a trend toward greater pre–post increases in the active tACS group compared to sham, alongside a significant between-group effect of Protocol. Although within-subject effects did not reach conventional significance, the convergence of these findings points to a context-dependent modulation of frontal alpha activity during active stress coping.

Functionally, increased frontal alpha power during stress exposure has been associated with inhibitory control and regulation of emotional information, potentially reflecting more efficient top–down modulation of stress responses. The alignment between this EEG pattern and the reduction in state anxiety observed behaviorally supports the interpretation that tACS may influence regulatory neural processes engaged during stress, even if these effects are subtle and variable.

Compared to resting-state EEG, task-based measures may thus offer greater sensitivity to stress-related neuromodulatory effects, an idea increasingly supported by recent EEG research emphasizing the importance of context and engagement (Kosonogov et al., 2024; Wang et al., 2024).

Autonomic indices derived from EDA and pupillometry showed consistent main effects of Time, reflecting changes in model fit, error, and slope parameters from pre to post-intervention. However, these changes did not differ between active and sham tACS conditions, indicating that they are more likely attributable to habituation or learning effects associated with repeated exposure to the stress paradigm rather than to neuromodulation.

A similar pattern was observed in our previous HB-tDCS study, where autonomic measures showed limited and inconsistent modulation despite repeated stimulation. Together, these findings suggest that autonomic stress markers may be relatively resistant to modulation in healthy individuals, or that larger samples, more vulnerable populations, or more intensive protocols are required to detect reliable effects.

When considered jointly, the results of the tACS study point to a selective and modest modulation of stress-related processes. The most robust effect was observed at the subjective affective level, with a specific reduction in state anxiety following active tACS. Neurophysiological effects were context-dependent, emerging more clearly during task engagement than at rest, while autonomic markers primarily reflected non-specific temporal effects.

From a resilience perspective, these findings are consistent with models proposing that early neuromodulatory effects may first manifest in subjective experience and cognitive–emotional regulation, with more stable physiological reorganization requiring repeated stimulation, higher baseline vulnerability, or multimodal interventions. In this sense, tACS may act as a facilitator of adaptive stress regulation, rather than as a direct driver of large-scale physiological change in healthy populations.

Several limitations should be considered when interpreting these findings. First, the use of a healthy, low-symptom sample may have constrained effect sizes due to floor effects. Second, sample sizes for physiological analyses were reduced by technical exclusions, limiting statistical power. Third, the use of a single-session tACS protocol, while appropriate for examining acute effects, may be insufficient to induce robust changes in more stable stress-related dimensions.

Finally, psychophysiological measures such as EDA and pupillometry are inherently noisy and sensitive to multiple sources of variability, which may obscure subtle neuromodulatory effects despite rigorous preprocessing.

In summary, this study shows that a single session of alpha-frequency tACS applied over fronto-parietal regions can induce an acute reduction in state anxiety following stress exposure, relative to sham stimulation. Neurophysiological and autonomic measures primarily reflected global temporal effects, with exploratory evidence suggesting that task-based frontal alpha activity may be more sensitive to tACS modulation than resting-state markers.

These findings complement our previous results and highlight the importance of stimulation modality, dosage, and measurement context when investigating neuromodulation effects on stress and resilience. Future studies should employ larger samples, repeated-session protocols, and multimodal designs to further elucidate the role of tACS in enhancing adaptive anxiety and stress regulation.

## Figures and Tables

**Table 1:** Sociodemographic characteristics of the tACS study samples.

Variable	Active tACS (n = 22)	Sham tACS (n = 20)
Age (years)	57.57 (7.51)	60.58 (7.32)
Sex		
Male	65.22%	57.89%
Female	34.78%	42.11%
Educational level		
Primary (≤ 8 years)	0.00%	4.35%
Secondary (9–12 years)	17.39%	21.73%
Higher education (≥ 13 years)	82.61%	73.92%

**Table 2:** Repeated-measures ANOVA results for each EDA and Pupil diameter measure. tACS and sham tACS are compared (R^2^, log MSE, Beta).

Metric	Variable (DV)	Source	SS	DF	MS	F	p-unc	η^2^g	ε
**EDA**	**R** ^**2**^	Time (PrePost)	0.020	1	0.020	4.582	**0.041**	0.051	1
		Error(Time)	0.128	29	0.004				1
		Condition (Real vs Sham)	0.004	1	0.004	1.021	0.321	0.035	
		Error(Between)	0.120	28	0.004				
		Time * Condition	0.000	1	0.000	0.010	0.923	0.000	1
		Error(Interaction)	0.256	28	0.009				1
	**MSE (log)**	Time (PrePost)	42.838	1	42.838	31.514	**0.000**	0.154	1
		Error(Time)	39.421	29	1.359				1
		Condition (Real vs Sham)	0.536	1	0.536	0.155	0.697	0.005	
		Error(Between)	96.994	28	3.464				
		Time * Condition	0.002	1	0.002	0.001	0.980	0.000	1
		Error(Interaction)	78.840	28	2.816				1
	**Beta (EST)**	Time (PrePost)	0.172	1	0.172	13.626	**0.001**	0.102	1
		Error(Time)	0.367	29	0.013				1
		Condition (Real vs Sham)	0.041	1	0.041	2.123	0.156	0.070	
		Error(Between)	0.538	28	0.019				
		Time * Condition	0.066	1	0.066	2.765	0.107	0.090	1
		Error(Interaction)	0.668	28	0.024				1
**Pupil diameter**	**Pupil R** ^**2**^	Time (PrePost)	0.023	1	0.023	4.385	**0.045**	0.032	1
		Error(Time)	0.153	29	0.005				1
		Condition (Real vs Sham)	0.027	1	0.027	2.988	0.095	0.096	
		Error(Between)	0.252	28	0.009				
		Time * Condition	0.000	1	0.000	0.005	0.942	0.000	1
		Error(Interaction)	0.307	28	0.011				1
	**Pupil MSE**	Time (PrePost)	0.007	1	0.007	0.392	0.536	0.006	1
		Error(Time)	0.527	29	0.018				1
		Condition (Real vs Sham)	0.003	1	0.003	0.283	0.599	0.010	
		Error(Between)	0.336	28	0.012				
		Time * Condition	0.033	1	0.033	0.902	0.350	0.031	1
		Error(Interaction)	1.021	28	0.036				1
	**Pupil Beta (EST)**	Time (PrePost)	0.610	1	0.610	5.313	**0.029**	0.035	1
		Error(Time)	3.331	29	0.115				1
		Condition (Real vs Sham)	0.569	1	0.569	2.540	0.122	0.083	
		Error(Between)	6.277	28	0.224				
		Time * Condition	0.037	1	0.037	0.156	0.696	0.006	1
		Error(Interaction)	6.624	28	0.237				1
